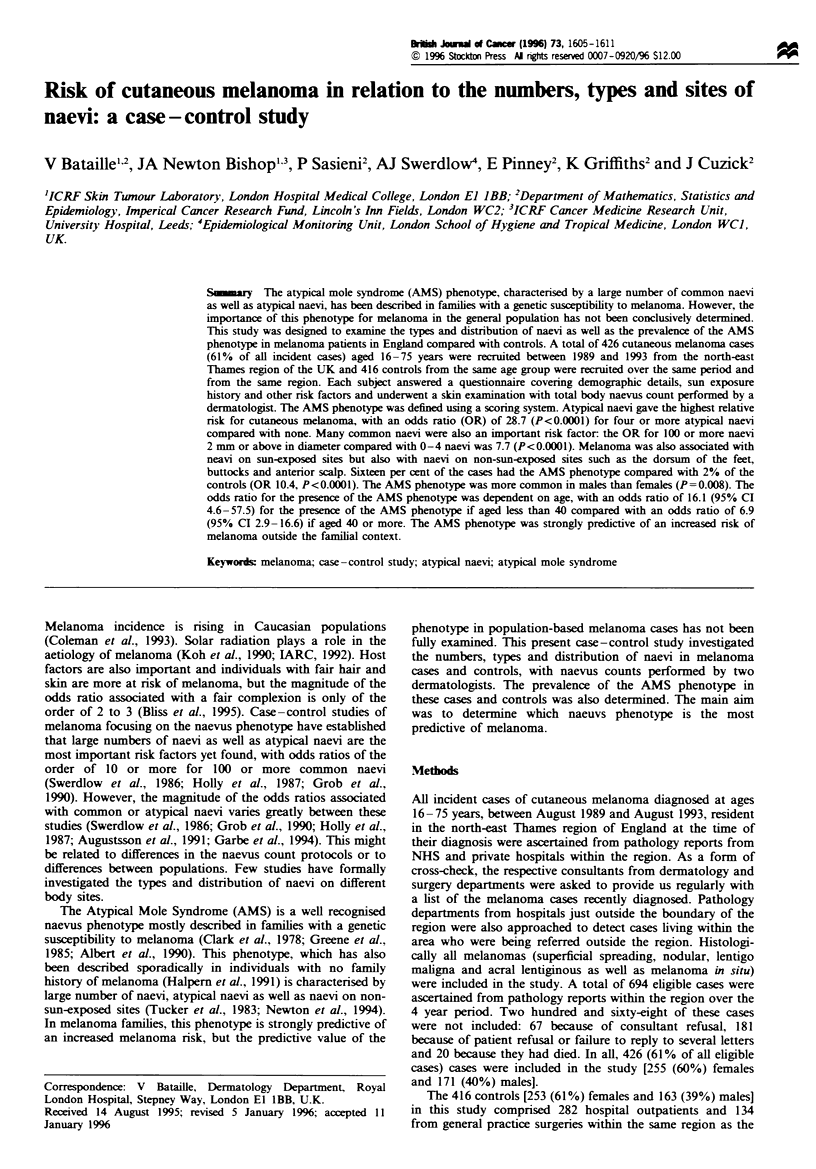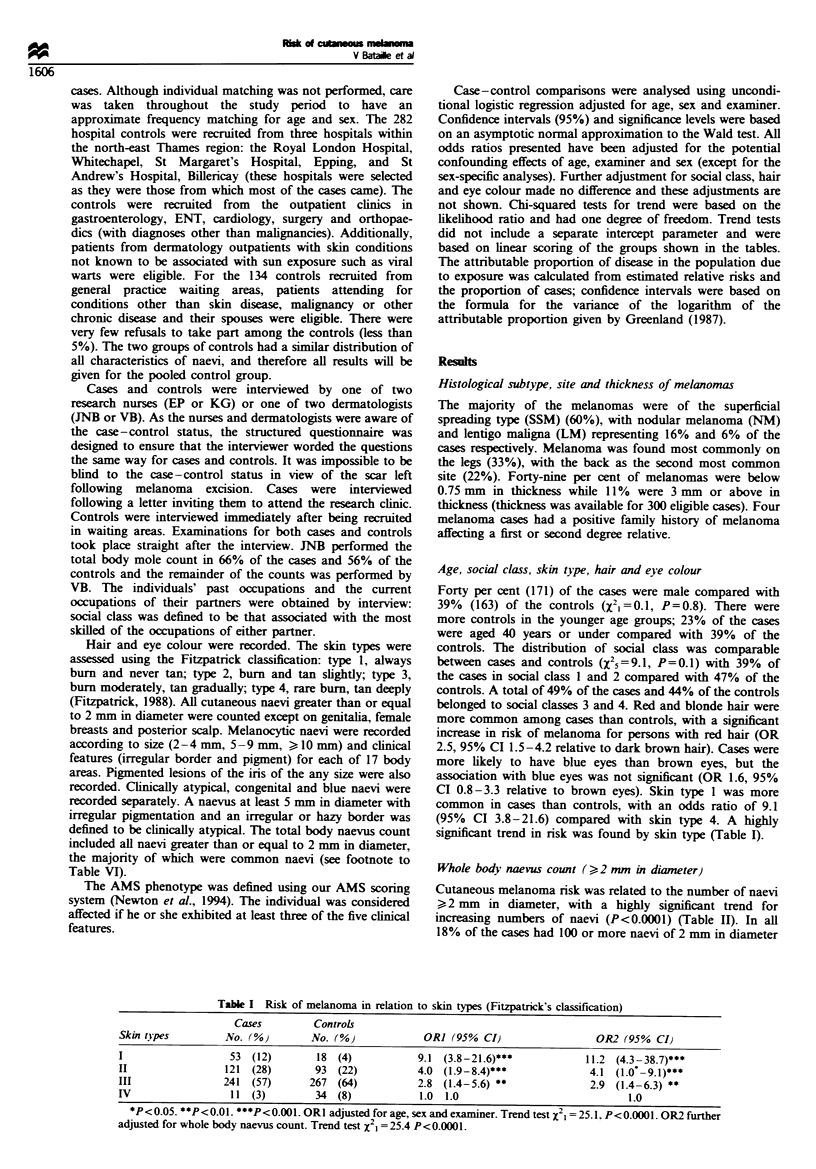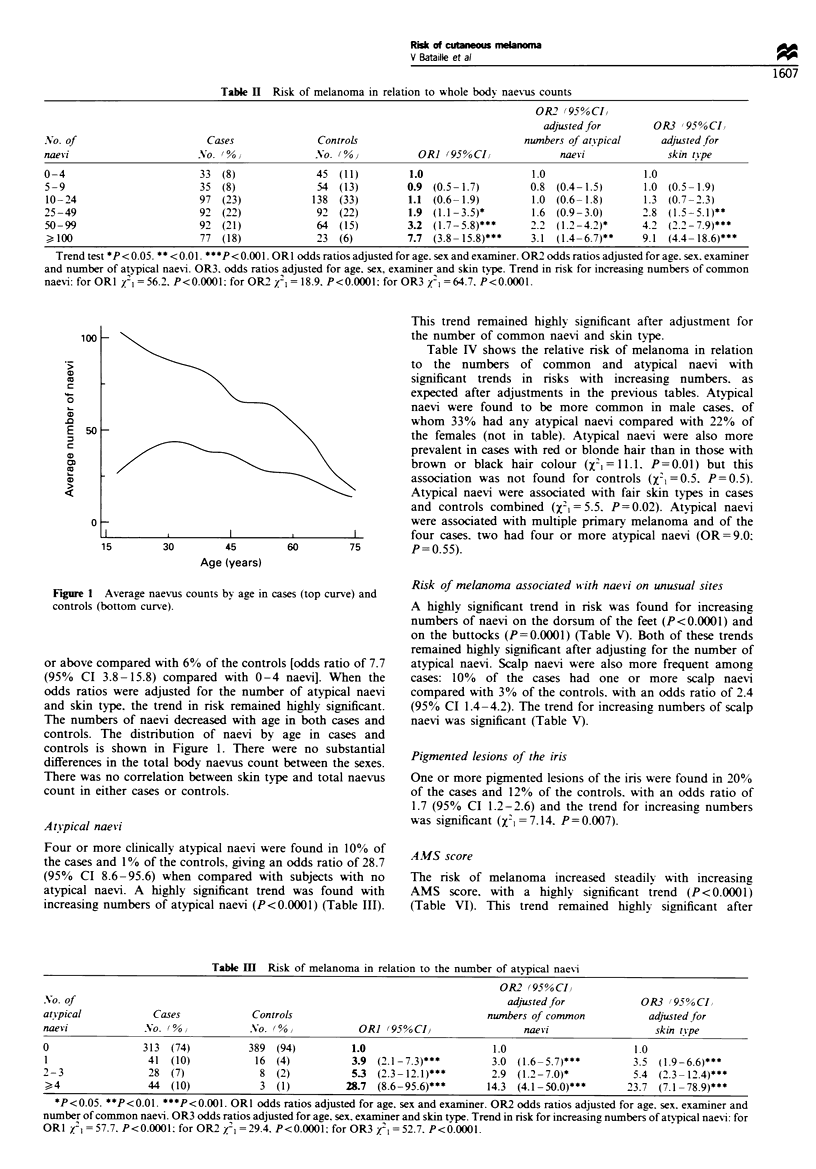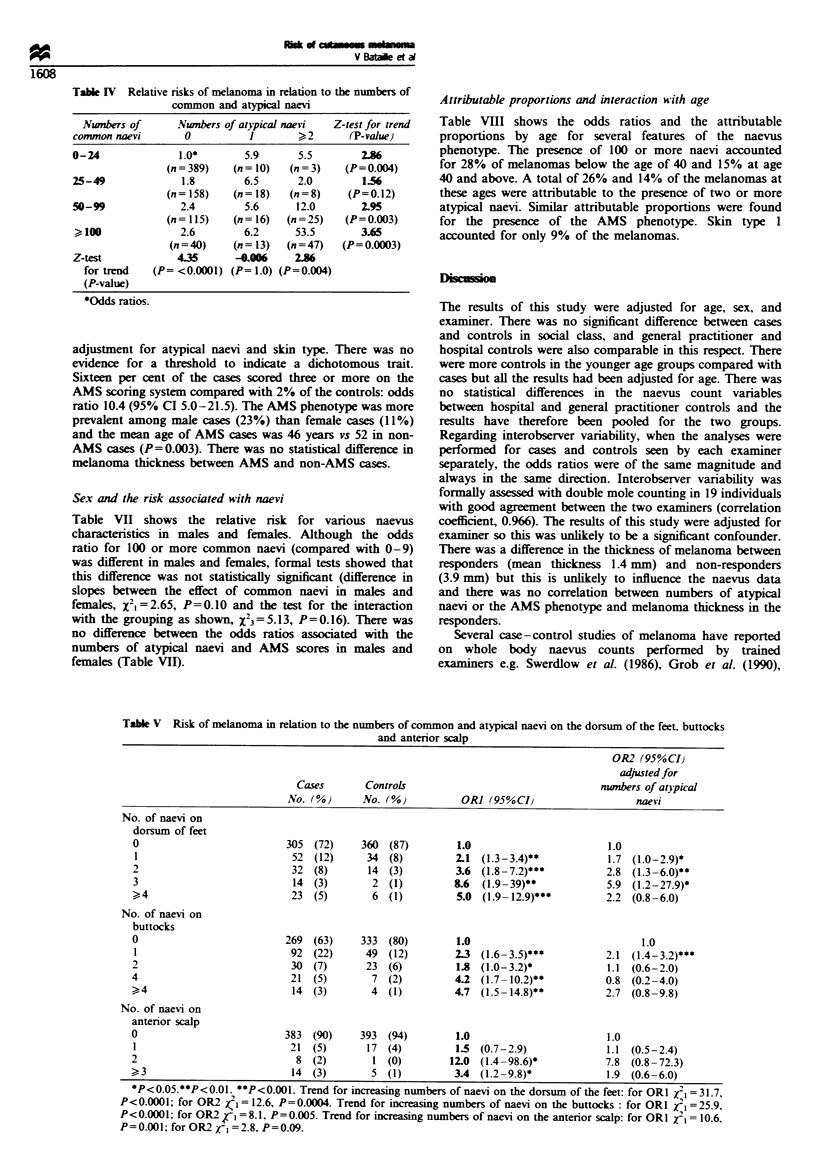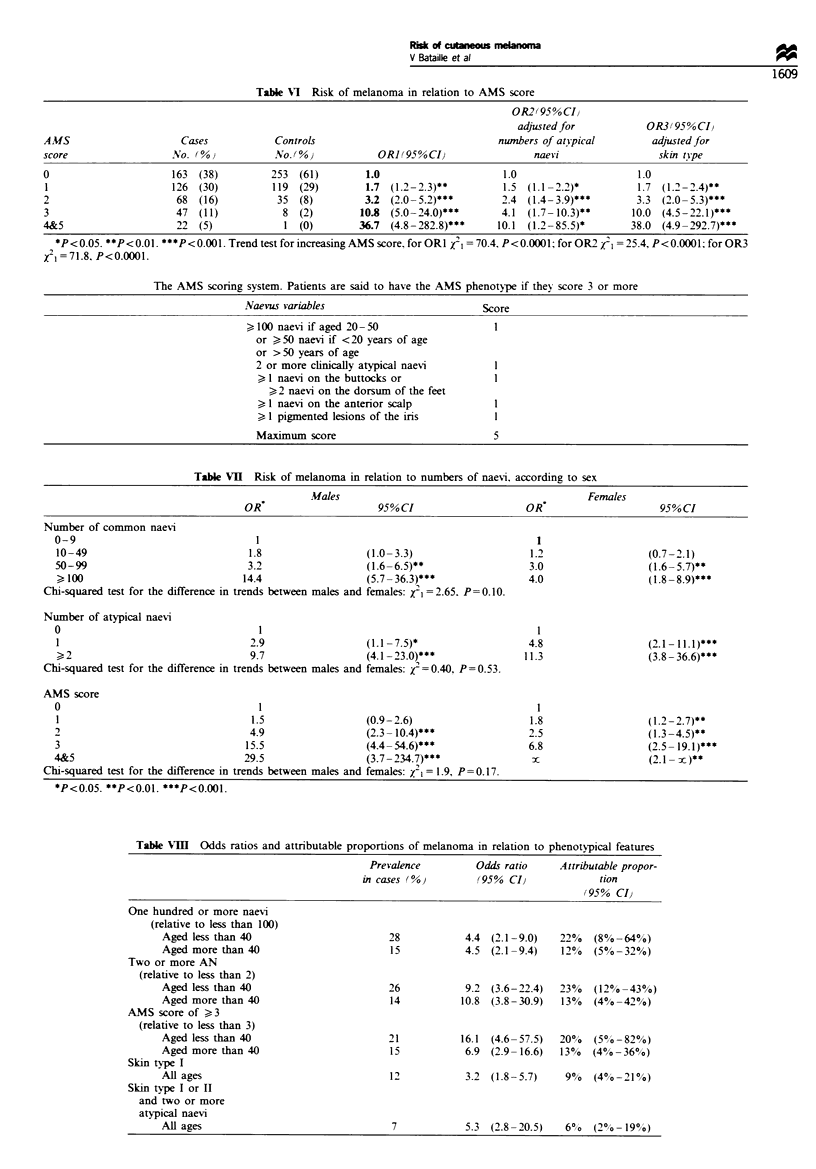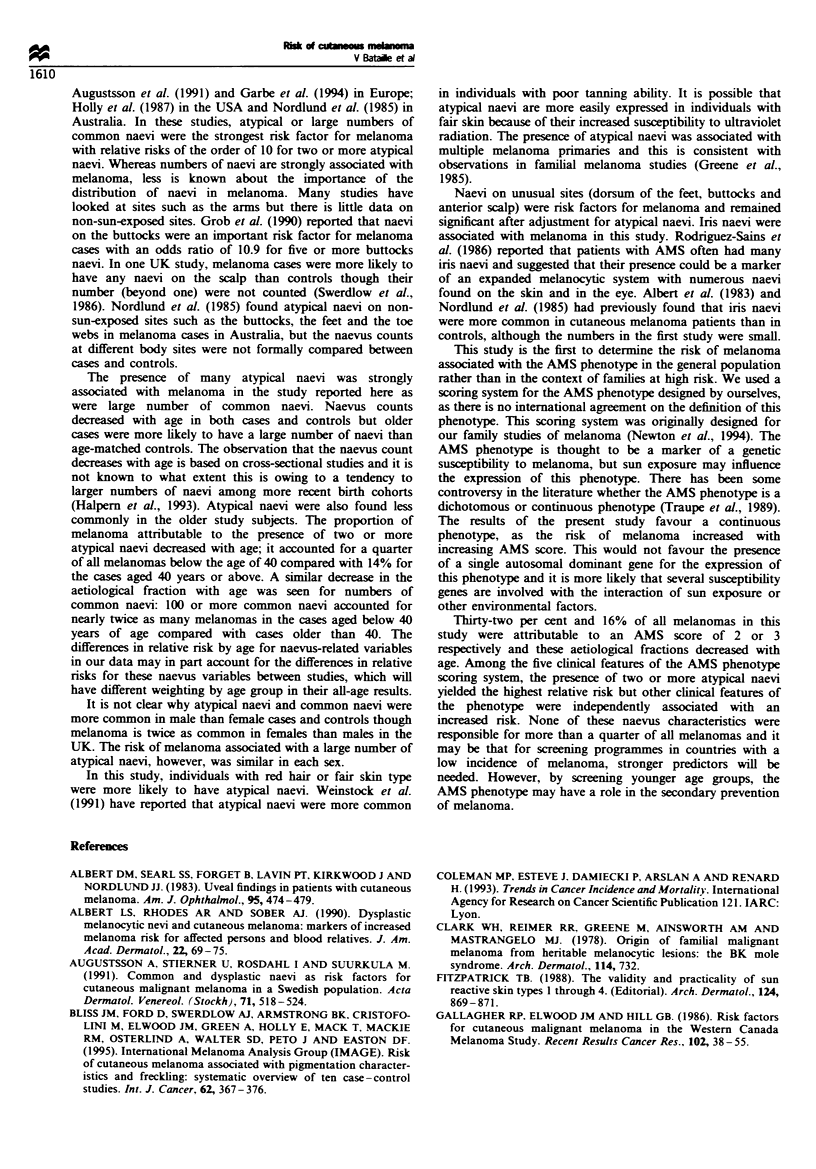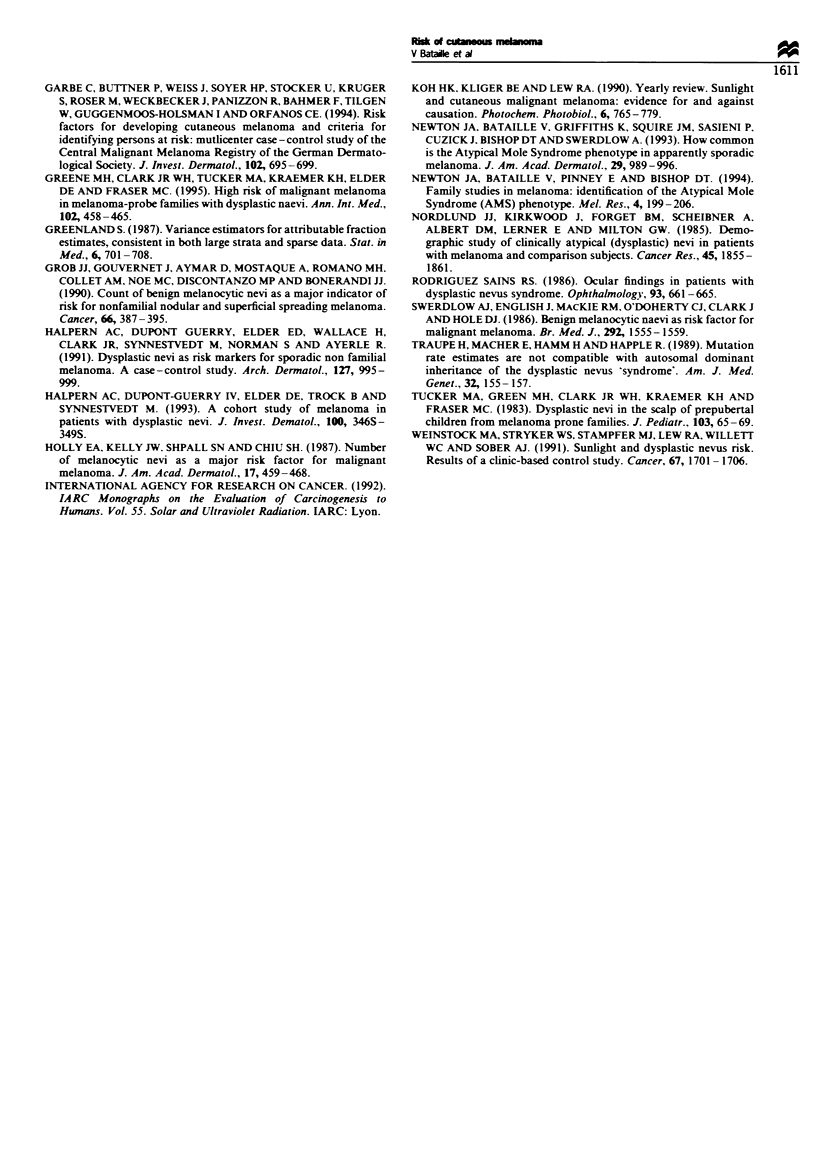# Risk of cutaneous melanoma in relation to the numbers, types and sites of naevi: a case-control study.

**DOI:** 10.1038/bjc.1996.302

**Published:** 1996-06

**Authors:** V. Bataille, J. A. Bishop, P. Sasieni, A. J. Swerdlow, E. Pinney, K. Griffiths, J. Cuzick

**Affiliations:** ICRF Skin Tumour Laboratory, London Hospital Medical College, UK.

## Abstract

The atypical mole syndrome (AMS) phenotype, characterised by a large number of common naevi as well as atypical naevi, has been described in families with a genetic susceptibility to melanoma. However, the importance of this phenotype for melanoma in the general population has not been conclusively determined. This study was designed to examine the types and distribution of naevi as well as the prevalence of the AMS phenotype in melanoma patients in England compared with controls. A total of 426 cutaneous melanoma cases (61% of all incident cases) aged 16-75 years were recruited between 1989 and 1993 from the north-east Thames region of the UK and 416 controls from the same age group were recruited over the same period and from the same region. Each subject answered a questionnaire covering demographic details, sun exposure history and other risk factors and underwent a skin examination with total body naevus count performed by a dermatologist. The AMS phenotype was defined using a scoring system. Atypical naevi gave the highest relative risk for cutaneous melanoma, with an odds ratio (OR) of 28.7 (P < 0.0001) for four or more atypical naevi compared with none. Many common naevi were also an important risk factor: the OR for 100 or more naevi 2 mm or above in diameter compared with 0-4 naevi was 7.7 (P < 0.0001). Melanoma was also associated with naevi on sun-exposed sites but also with naevi on non-sun-exposed sites such as the dorsum of the feet, buttocks and anterior scalp. Sixteen per cent of the cases had the AMS phenotype compared with 2% of the controls (OR 10.4, P < 0.0001). The AMS phenotype was more common in males than females (P = 0.008). The odds ratio for the presence of the AMS phenotype was dependent on age, with an odds ratio of 16.1 (95% CI 4.6-57.5) for the presence of the AMS phenotype if aged less than 40 compared with an odds ratio of 6.9 (95% CI 2.9-16.6) if aged 40 or more. The AMS phenotype was strongly predictive of an increased risk of melanoma outside the familial context.


British Journal of Cancer (1996) 73, 1605-1611

© 1996 Stockton Press All rights reserved 0007-0920/96 $12.00

### Risk of cutaneous melanoma in relation to the numbers, types and sites of naevi: a case-control study

V Bataille[1,2], JA Newton Bishop[1,3], P Sasieni[2], AJ Swerdlow[4], E Pinney[2], K Griffiths[2] and J Cuzick[2]

[1]*ICRF Skin Tumour Laboratory, London Hospital Medical College, London E1 1BB;* [2]*Department of Mathematics, Statistics and Epidemiology, Imperical Cancer Research Fund, Lincoln's Inn Fields, London WC2;* [3]*ICRF Cancer Medicine Research Unit, University Hospital, Leeds;* [4]*Epidemiological Monitoring Unit, London School of Hygiene and Tropical Medicine, London WC1, UK.*

**Summary** The atypical mole syndrome (AMS) phenotype, characterised by a large number of common naevi as well as atypical naevi, has been described in families with a genetic susceptibility to melanoma. However, the importance of this phenotype for melanoma in the general population has not been conclusively determined. This study was designed to examine the types and distribution of naevi as well as the prevalence of the AMS phenotype in melanoma patients in England compared with controls. A total of 426 cutaneous melanoma cases (61% of all incident cases) aged 16-75 years were recruited between 1989 and 1993 from the north-east Thames region of the UK and 416 controls from the same age group were recruited over the same period and from the same region. Each subject answered a questionnaire covering demographic details, sun exposure history and other risk factors and underwent a skin examination with total body naevus count performed by a dermatologist. The AMS phenotype was defined using a scoring system. Atypical naevi gave the highest relative risk for cutaneous melanoma, with an odds ratio (OR) of 28.7 ($P<0.0001$) for four or more atypical naevi compared with none. Many common naevi were also an important risk factor: the OR for 100 or more naevi 2 mm or above in diameter compared with 0-4 naevi was 7.7 ($P<0.0001$). Melanoma was also associated with neavi on sun-exposed sites but also with naevi on non-sun-exposed sites such as the dorsum of the feet, buttocks and anterior scalp. Sixteen per cent of the cases had the AMS phenotype compared with 2% of the controls (OR 10.4, $P<0.0001$). The AMS phenotype was more common in males than females ($P=0.008$). The odds ratio for the presence of the AMS phenotype was dependent on age, with an odds ratio of 16.1 (95% CI 4.6-57.5) for the presence of the AMS phenotype if aged less than 40 compared with an odds ratio of 6.9 (95% CI 2.9-16.6) if aged 40 or more. The AMS phenotype was strongly predictive of an increased risk of melanoma outside the familial context.

**Keywords:** melanoma; case-control study; atypical naevi; atypical mole syndrome

Melanoma incidence is rising in Caucasian populations (Coleman *et al.*, 1993). Solar radiation plays a role in the aetiology of melanoma (Koh *et al.*, 1990; IARC, 1992). Host factors are also important and individuals with fair hair and skin are more at risk of melanoma, but the magnitude of the odds ratio associated with a fair complexion is only of the order of 2 to 3 (Bliss *et al.*, 1995). Case-control studies of melanoma focusing on the naevus phenotype have established that large numbers of naevi as well as atypical naevi are the most important risk factors yet found, with odds ratios of the order of 10 or more for 100 or more common naevi (Swerdlow *et al.*, 1986; Holly *et al.*, 1987; Grob *et al.*, 1990). However, the magnitude of the odds ratios associated with common or atypical naevi varies greatly between these studies (Swerdlow *et al.*, 1986; Grob *et al.*, 1990; Holly *et al.*, 1987; Augustsson *et al.*, 1991; Garbe *et al.*, 1994). This might be related to differences in the naevus count protocols or to differences between populations. Few studies have formally investigated the types and distribution of naevi on different body sites.

The Atypical Mole Syndrome (AMS) is a well recognised naevus phenotype mostly described in families with a genetic susceptibility to melanoma (Clark *et al.*, 1978; Greene *et al.*, 1985; Albert *et al.*, 1990). This phenotype, which has also been described sporadically in individuals with no family history of melanoma (Halpern *et al.*, 1991) is characterised by large number of naevi, atypical naevi as well as naevi on non-sun-exposed sites (Tucker *et al.*, 1983; Newton *et al.*, 1994). In melanoma families, this phenotype is strongly predictive of an increased melanoma risk, but the predictive value of the phenotype in population-based melanoma cases has not been fully examined. This present case-control study investigated the numbers, types and distribution of naevi in melanoma cases and controls, with naevus counts performed by two dermatologists. The prevalence of the AMS phenotype in these cases and controls was also determined. The main aim was to determine which naeuvs phenotype is the most predictive of melanoma.

### Methods

All incident cases of cutaneous melanoma diagnosed at ages 16-75 years, between August 1989 and August 1993, resident in the north-east Thames region of England at the time of their diagnosis were ascertained from pathology reports from NHS and private hospitals within the region. As a form of cross-check, the respective consultants from dermatology and surgery departments were asked to provide us regularly with a list of the melanoma cases recently diagnosed. Pathology departments from hospitals just outside the boundary of the region were also approached to detect cases living within the area who were being referred outside the region. Histologically all melanomas (superficial spreading, nodular, lentigo maligna and acral lentiginous as well as melanoma *in situ*) were included in the study. A total of 694 eligible cases were ascertained from pathology reports within the region over the 4 year period. Two hundred and sixty-eight of these cases were not included: 67 because of consultant refusal, 181 because of patient refusal or failure to reply to several letters and 20 because they had died. In all, 426 (61% of all eligible cases) cases were included in the study [255 (60%) females and 171 (40%) males].

The 416 controls [253 (61%) females and 163 (39%) males] in this study comprised 282 hospital outpatients and 134 from general practice surgeries within the same region as the

Correspondence: V Bataille, Dermatology Department, Royal London Hospital, Stepney Way, London E1 1BB, U.K.
Received 14 August 1995; revised 5 January 1996; accepted 11 January 1996

cases. Although individual matching was not performed, care was taken throughout the study period to have an approximate frequency matching for age and sex. The 282 hospital controls were recruited from three hospitals within the north-east Thames region: the Royal London Hospital, Whitechapel, St Margaret's Hospital, Epping, and St Andrew's Hospital, Billericay (these hospitals were selected as they were those from which most of the cases came). The controls were recruited from the outpatient clinics in gastroenterology, ENT, cardiology, surgery and orthopaedics (with diagnoses other than malignancies). Additionally, patients from dermatology outpatients with skin conditions not known to be associated with sun exposure such as viral warts were eligible. For the 134 controls recruited from general practice waiting areas, patients attending for conditions other than skin disease, malignancy or other chronic disease and their spouses were eligible. There were very few refusals to take part among the controls (less than 5%). The two groups of controls had a similar distribution of all characteristics of naevi, and therefore all results will be given for the pooled control group.

Cases and controls were interviewed by one of two research nurses (EP or KG) or one of two dermatologists (JNB or VB). As the nurses and dermatologists were aware of the case–control status, the structured questionnaire was designed to ensure that the interviewer worded the questions the same way for cases and controls. It was impossible to be blind to the case–control status in view of the scar left following melanoma excision. Cases were interviewed following a letter inviting them to attend the research clinic. Controls were interviewed immediately after being recruited in waiting areas. Examinations for both cases and controls took place straight after the interview. JNB performed the total body mole count in 66% of the cases and 56% of the controls and the remainder of the counts was performed by VB. The individuals' past occupations and the current occupations of their partners were obtained by interview: social class was defined to be that associated with the most skilled of the occupations of either partner.

Hair and eye colour were recorded. The skin types were assessed using the Fitzpatrick classification: type 1, always burn and never tan; type 2, burn and tan slightly; type 3, burn moderately, tan gradually; type 4, rare burn, tan deeply (Fitzpatrick, 1988). All cutaneous naevi greater than or equal to 2 mm in diameter were counted except on genitalia, female breasts and posterior scalp. Melanocytic naevi were recorded according to size (2–4 mm, 5–9 mm, $\geqslant$10 mm) and clinical features (irregular border and pigment) for each of 17 body areas. Pigmented lesions of the iris of the any size were also recorded. Clinically atypical, congenital and blue naevi were recorded separately. A naevus at least 5 mm in diameter with irregular pigmentation and an irregular or hazy border was defined to be clinically atypical. The total body naevus count included all naevi greater than or equal to 2 mm in diameter, the majority of which were common naevi (see footnote to Table VI).

The AMS phenotype was defined using our AMS scoring system (Newton *et al.*, 1994). The individual was considered affected if he or she exhibited at least three of the five clinical features.

Case–control comparisons were analysed using unconditional logistic regression adjusted for age, sex and examiner. Confidence intervals (95%) and significance levels were based on an asymptotic normal approximation to the Wald test. All odds ratios presented have been adjusted for the potential confounding effects of age, examiner and sex (except for the sex-specific analyses). Further adjustment for social class, hair and eye colour made no difference and these adjustments are not shown. Chi-squared tests for trend were based on the likelihood ratio and had one degree of freedom. Trend tests did not include a separate intercept parameter and were based on linear scoring of the groups shown in the tables. The attributable proportion of disease in the population due to exposure was calculated from estimated relative risks and the proportion of cases; confidence intervals were based on the formula for the variance of the logarithm of the attributable proportion given by Greenland (1987).

### Results

### *Histological subtype, site and thickness of melanomas*

The majority of the melanomas were of the superficial spreading type (SSM) (60%), with nodular melanoma (NM) and lentigo maligna (LM) representing 16% and 6% of the cases respectively. Melanoma was found most commonly on the legs (33%), with the back as the second most common site (22%). Forty-nine per cent of melanomas were below 0.75 mm in thickness while 11% were 3 mm or above in thickness (thickness was available for 300 eligible cases). Four melanoma cases had a positive family history of melanoma affecting a first or second degree relative.

### *Age, social class, skin type, hair and eye colour*

Forty per cent (171) of the cases were male compared with 39% (163) of the controls ($\chi^2_1=0.1$, $P=0.8$). There were more controls in the younger age groups; 23% of the cases were aged 40 years or under compared with 39% of the controls. The distribution of social class was comparable between cases and controls ($\chi^2_5=9.1$, $P=0.1$) with 39% of the cases in social class 1 and 2 compared with 47% of the controls. A total of 49% of the cases and 44% of the controls belonged to social classes 3 and 4. Red and blonde hair were more common among cases than controls, with a significant increase in risk of melanoma for persons with red hair (OR 2.5, 95% CI 1.5–4.2 relative to dark brown hair). Cases were more likely to have blue eyes than brown eyes, but the association with blue eyes was not significant (OR 1.6, 95% CI 0.8–3.3 relative to brown eyes). Skin type 1 was more common in cases than controls, with an odds ratio of 9.1 (95% CI 3.8–21.6) compared with skin type 4. A highly significant trend in risk was found by skin type (Table I).

### *Whole body naevus count ($\geqslant$2 mm in diameter)*

Cutaneous melanoma risk was related to the number of naevi $\geqslant$2 mm in diameter, with a highly significant trend for increasing numbers of naevi ($P<0.0001$) (Table II). In all 18% of the cases had 100 or more naevi of 2 mm in diameter

**Table I** Risk of melanoma in relation to skin types (Fitzpatrick's classification)

| *Skin types* | *Cases* *No. (%)* | *Controls* *No. (%)* | *OR1 (95% CI)* | *OR2 (95% CI)* |
|---|---|---|---|---|
| I | 53 (12) | 18 (4) | 9.1 (3.8–21.6)*** | 11.2 (4.3–38.7)*** |
| II | 121 (28) | 93 (22) | 4.0 (1.9–8.4)*** | 4.1 (1.0*–9.1)*** |
| III | 241 (57) | 267 (64) | 2.8 (1.4–5.6) ** | 2.9 (1.4–6.3) ** |
| IV | 11 (3) | 34 (8) | 1.0 1.0 | 1.0 |

*$P<0.05$. **$P<0.01$. ***$P<0.001$. OR1 adjusted for age, sex and examiner. Trend test $\chi^2_1=25.1$, $P<0.0001$. OR2 further adjusted for whole body naevus count. Trend test $\chi^2_1=25.4$ $P<0.0001$.

**Table II** Risk of melanoma in relation to whole body naevus counts

| *No. of naevi* | *Cases No. (%)* | *Controls No. (%)* | *OR1 (95%CI)* | *OR2 (95%CI) adjusted for numbers of atypical naevi* | *OR3 (95%CI) adjusted for skin type* |
|---|---|---|---|---|---|
| 0-4 | 33 (8) | 45 (11) | **1.0** | 1.0 | 1.0 |
| 5-9 | 35 (8) | 54 (13) | **0.9** (0.5-1.7) | 0.8 (0.4-1.5) | 1.0 (0.5-1.9) |
| 10-24 | 97 (23) | 138 (33) | **1.1** (0.6-1.9) | 1.0 (0.6-1.8) | 1.3 (0.7-2.3) |
| 25-49 | 92 (22) | 92 (22) | **1.9** (1.1-3.5)* | 1.6 (0.9-3.0) | 2.8 (1.5-5.1)** |
| 50-99 | 92 (21) | 64 (15) | **3.2** (1.7-5.8)*** | 2.2 (1.2-4.2)* | 4.2 (2.2-7.9)*** |
| ≥100 | 77 (18) | 23 (6) | **7.7** (3.8-15.8)*** | 3.1 (1.4-6.7)** | 9.1 (4.4-18.6)*** |

Trend test *$P<0.05$. **$<0.01$. ***$P<0.001$. OR1 odds ratios adjusted for age, sex and examiner. OR2 odds ratios adjusted for age, sex, examiner and number of atypical naevi. OR3, odds ratios adjusted for age, sex, examiner and skin type. Trend in risk for increasing numbers of common naevi: for OR1 $\chi^2_1=56.2$, $P<0.0001$; for OR2 $\chi^2_1=18.9$, $P<0.0001$; for OR3 $\chi^2_1=64.7$, $P<0.0001$.

**Figure 1** Average naevus counts by age in cases (top curve) and controls (bottom curve).

or above compared with 6% of the controls [odds ratio of 7.7 (95% CI 3.8-15.8) compared with 0-4 naevi]. When the odds ratios were adjusted for the number of atypical naevi and skin type, the trend in risk remained highly significant. The numbers of naevi decreased with age in both cases and controls. The distribution of naevi by age in cases and controls is shown in Figure 1. There were no substantial differences in the total body naevus count between the sexes. There was no correlation between skin type and total naevus count in either cases or controls.

### *Atypical naevi*

Four or more clinically atypical naevi were found in 10% of the cases and 1% of the controls, giving an odds ratio of 28.7 (95% CI 8.6-95.6) when compared with subjects with no atypical naevi. A highly significant trend was found with increasing numbers of atypical naevi ($P<0.0001$) (Table III). This trend remained highly significant after adjustment for the number of common naevi and skin type.

Table IV shows the relative risk of melanoma in relation to the numbers of common and atypical naevi with significant trends in risks with increasing numbers, as expected after adjustments in the previous tables. Atypical naevi were found to be more common in male cases, of whom 33% had any atypical naevi compared with 22% of the females (not in table). Atypical naevi were also more prevalent in cases with red or blonde hair than in those with brown or black hair colour ($\chi^2_1=11.1$, $P=0.01$) but this association was not found for controls ($\chi^2_1=0.5$, $P=0.5$). Atypical naevi were associated with fair skin types in cases and controls combined ($\chi^2_1=5.5$, $P=0.02$). Atypical naevi were associated with multiple primary melanoma and of the four cases, two had four or more atypical naevi (OR=9.0; $P=0.55$).

### *Risk of melanoma associated with naevi on unusual sites*

A highly significant trend in risk was found for increasing numbers of naevi on the dorsum of the feet ($P<0.0001$) and on the buttocks ($P=0.0001$) (Table V). Both of these trends remained highly significant after adjusting for the number of atypical naevi. Scalp naevi were also more frequent among cases: 10% of the cases had one or more scalp naevi compared with 3% of the controls, with an odds ratio of 2.4 (95% CI 1.4-4.2). The trend for increasing numbers of scalp naevi was significant (Table V).

### *Pigmented lesions of the iris*

One or more pigmented lesions of the iris were found in 20% of the cases and 12% of the controls, with an odds ratio of 1.7 (95% CI 1.2-2.6) and the trend for increasing numbers was significant ($\chi^2_1=7.14$, $P=0.007$).

### *AMS score*

The risk of melanoma increased steadily with increasing AMS score, with a highly significant trend ($P<0.0001$) (Table VI). This trend remained highly significant after

**Table III** Risk of melanoma in relation to the number of atypical naevi

| *No. of atypical naevi* | *Cases No. (%)* | *Controls No. (%)* | *OR1 (95%CI)* | *OR2 (95%CI) adjusted for numbers of common naevi* | *OR3 (95%CI) adjusted for skin type* |
|---|---|---|---|---|---|
| 0 | 313 (74) | 389 (94) | **1.0** | 1.0 | 1.0 |
| 1 | 41 (10) | 16 (4) | **3.9** (2.1-7.3)*** | 3.0 (1.6-5.7)*** | 3.5 (1.9-6.6)*** |
| 2-3 | 28 (7) | 8 (2) | **5.3** (2.3-12.1)*** | 2.9 (1.2-7.0)* | 5.4 (2.3-12.4)*** |
| ≥4 | 44 (10) | 3 (1) | **28.7** (8.6-95.6)*** | 14.3 (4.1-50.0)*** | 23.7 (7.1-78.9)*** |

*$P<0.05$. **$P<0.01$. ***$P<0.001$. OR1 odds ratios adjusted for age, sex and examiner. OR2 odds ratios adjusted for age, sex, examiner and number of common naevi. OR3 odds ratios adjusted for age, sex, examiner and skin type. Trend in risk for increasing numbers of atypical naevi: for OR1 $\chi^2_1=57.7$, $P<0.0001$; for OR2 $\chi^2_1=29.4$, $P<0.0001$; for OR3 $\chi^2_1=52.7$, $P<0.0001$.

Table IV Relative risks of melanoma in relation to the numbers of common and atypical naevi

| Numbers of common naevi | Numbers of atypical naevi 0 | 1 | ≥2 | Z-test for trend (P-value) |
|---|---|---|---|---|
| **0–24** | 1.0* ($n=389$) | 5.9 ($n=10$) | 5.5 ($n=3$) | **2.86** ($P=0.004$) |
| **25–49** | 1.8 ($n=158$) | 6.5 ($n=18$) | 2.0 ($n=8$) | **1.56** ($P=0.12$) |
| **50–99** | 2.4 ($n=115$) | 5.6 ($n=16$) | 12.0 ($n=25$) | **2.95** ($P=0.003$) |
| **≥100** | 2.6 ($n=40$) | 6.2 ($n=13$) | 53.5 ($n=47$) | **3.65** ($P=0.0003$) |
| Z-test for trend (P-value) | **4.35** ($P=<0.0001$) | **−0.006** ($P=1.0$) | **2.86** ($P=0.004$) | |

*Odds ratios.

adjustment for atypical naevi and skin type. There was no evidence for a threshold to indicate a dichotomous trait. Sixteen per cent of the cases scored three or more on the AMS scoring system compared with 2% of the controls: odds ratio 10.4 (95% CI 5.0–21.5). The AMS phenotype was more prevalent among male cases (23%) than female cases (11%) and the mean age of AMS cases was 46 years *vs* 52 in non-AMS cases ($P=0.003$). There was no statistical difference in melanoma thickness between AMS and non-AMS cases.

### *Sex and the risk associated with naevi*

Table VII shows the relative risk for various naevus characteristics in males and females. Although the odds ratio for 100 or more common naevi (compared with 0–9) was different in males and females, formal tests showed that this difference was not statistically significant (difference in slopes between the effect of common naevi in males and females, $\chi^2_1=2.65$, $P=0.10$ and the test for the interaction with the grouping as shown, $\chi^2_3=5.13$, $P=0.16$). There was no difference between the odds ratios associated with the numbers of atypical naevi and AMS scores in males and females (Table VII).

### *Attributable proportions and interaction with age*

Table VIII shows the odds ratios and the attributable proportions by age for several features of the naevus phenotype. The presence of 100 or more naevi accounted for 28% of melanomas below the age of 40 and 15% at age 40 and above. A total of 26% and 14% of the melanomas at these ages were attributable to the presence of two or more atypical naevi. Similar attributable proportions were found for the presence of the AMS phenotype. Skin type 1 accounted for only 9% of the melanomas.

### Discussion

The results of this study were adjusted for age, sex, and examiner. There was no significant difference between cases and controls in social class, and general practitioner and hospital controls were also comparable in this respect. There were more controls in the younger age groups compared with cases but all the results had been adjusted for age. There was no statistical differences in the naevus count variables between hospital and general practitioner controls and the results have therefore been pooled for the two groups. Regarding interobserver variability, when the analyses were performed for cases and controls seen by each examiner separately, the odds ratios were of the same magnitude and always in the same direction. Interobserver variability was formally assessed with double mole counting in 19 individuals with good agreement between the two examiners (correlation coefficient, 0.966). The results of this study were adjusted for examiner so this was unlikely to be a significant confounder. There was a difference in the thickness of melanoma between responders (mean thickness 1.4 mm) and non-responders (3.9 mm) but this is unlikely to influence the naevus data and there was no correlation between numbers of atypical naevi or the AMS phenotype and melanoma thickness in the responders.

Several case–control studies of melanoma have reported on whole body naevus counts performed by trained examiners e.g. Swerdlow *et al.* (1986), Grob *et al.* (1990),

Table V Risk of melanoma in relation to the numbers of common and atypical naevi on the dorsum of the feet, buttocks and anterior scalp

| | Cases No. (%) | Controls No. (%) | OR1 (95%CI) | OR2 (95%CI) adjusted for numbers of atypical naevi |
|---|---|---|---|---|
| No. of naevi on dorsum of feet | | | | |
| 0 | 305 (72) | 360 (87) | **1.0** | 1.0 |
| 1 | 52 (12) | 34 (8) | **2.1** (1.3–3.4)** | 1.7 (1.0–2.9)* |
| 2 | 32 (8) | 14 (3) | **3.6** (1.8–7.2)*** | 2.8 (1.3–6.0)** |
| 3 | 14 (3) | 2 (1) | **8.6** (1.9–39)** | 5.9 (1.2–27.9)* |
| ≥4 | 23 (5) | 6 (1) | **5.0** (1.9–12.9)*** | 2.2 (0.8–6.0) |
| No. of naevi on buttocks | | | | |
| 0 | 269 (63) | 333 (80) | **1.0** | 1.0 |
| 1 | 92 (22) | 49 (12) | **2.3** (1.6–3.5)*** | 2.1 (1.4–3.2)*** |
| 2 | 30 (7) | 23 (6) | **1.8** (1.0–3.2)* | 1.1 (0.6–2.0) |
| 4 | 21 (5) | 7 (2) | **4.2** (1.7–10.2)** | 0.8 (0.2–4.0) |
| ≥4 | 14 (3) | 4 (1) | **4.7** (1.5–14.8)** | 2.7 (0.8–9.8) |
| No. of naevi on anterior scalp | | | | |
| 0 | 383 (90) | 393 (94) | **1.0** | 1.0 |
| 1 | 21 (5) | 17 (4) | **1.5** (0.7–2.9) | 1.1 (0.5–2.4) |
| 2 | 8 (2) | 1 (0) | **12.0** (1.4–98.6)* | 7.8 (0.8–72.3) |
| ≥3 | 14 (3) | 5 (1) | **3.4** (1.2–9.8)* | 1.9 (0.6–6.0) |

*$P<0.05$.**$P<0.01$. **$P<0.001$. Trend for increasing numbers of naevi on the dorsum of the feet: for OR1 $\chi^2_1=31.7$, $P<0.0001$; for OR2 $\chi^2_1=12.6$, $P=0.0004$. Trend for increasing numbers of naevi on the buttocks : for OR1 $\chi^2_1=25.9$, $P<0.0001$; for OR2 $\chi^2_1=8.1$, $P=0.005$. Trend for increasing numbers of naevi on the anterior scalp: for OR1 $\chi^2_1=10.6$, $P=0.001$; for OR2 $\chi^2_1=2.8$, $P=0.09$.

**Table VI** Risk of melanoma in relation to AMS score

| *AMS score* | *Cases No. (%)* | *Controls No.(%)* | *OR1(95%CI)* | *OR2(95%CI) adjusted for numbers of atypical naevi* | *OR3(95%CI) adjusted for skin type* |
|---|---|---|---|---|---|
| 0 | 163 (38) | 253 (61) | **1.0** | 1.0 | 1.0 |
| 1 | 126 (30) | 119 (29) | **1.7** (1.2-2.3)** | 1.5 (1.1-2.2)* | 1.7 (1.2-2.4)** |
| 2 | 68 (16) | 35 (8) | **3.2** (2.0-5.2)*** | 2.4 (1.4-3.9)*** | 3.3 (2.0-5.3)*** |
| 3 | 47 (11) | 8 (2) | **10.8** (5.0-24.0)*** | 4.1 (1.7-10.3)** | 10.0 (4.5-22.1)*** |
| 4&5 | 22 (5) | 1 (0) | **36.7** (4.8-282.8)*** | 10.1 (1.2-85.5)* | 38.0 (4.9-292.7)*** |

*$P<0.05$. **$P<0.01$. ***$P<0.001$. Trend test for increasing AMS score, for OR1 $\chi^2_1=70.4$, $P<0.0001$; for OR2 $\chi^2_1=25.4$, $P<0.0001$; for OR3 $\chi^2_1=71.8$, $P<0.0001$.

The AMS scoring system. Patients are said to have the AMS phenotype if they score 3 or more

| *Naevus variables* | Score |
|---|---|
| ≥100 naevi if aged 20-50 or ≥50 naevi if <20 years of age or >50 years of age | 1 |
| 2 or more clinically atypical naevi | 1 |
| ≥1 naevi on the buttocks or ≥2 naevi on the dorsum of the feet | 1 |
| ≥1 naevi on the anterior scalp | 1 |
| ≥1 pigmented lesions of the iris | 1 |
| Maximum score | 5 |

**Table VII** Risk of melanoma in relation to numbers of naevi, according to sex

| | *Males OR** | *Males 95%CI* | *Females OR** | *Females 95%CI* |
|---|---|---|---|---|
| Number of common naevi | | | | |
| 0-9 | 1 | | 1 | |
| 10-49 | 1.8 | (1.0-3.3) | 1.2 | (0.7-2.1) |
| 50-99 | 3.2 | (1.6-6.5)** | 3.0 | (1.6-5.7)** |
| ≥100 | 14.4 | (5.7-36.3)*** | 4.0 | (1.8-8.9)*** |
| Chi-squared test for the difference in trends between males and females: $\chi^2_1=2.65$, $P=0.10$. | | | | |
| Number of atypical naevi | | | | |
| 0 | 1 | | 1 | |
| 1 | 2.9 | (1.1-7.5)* | 4.8 | (2.1-11.1)*** |
| ≥2 | 9.7 | (4.1-23.0)*** | 11.3 | (3.8-36.6)*** |
| Chi-squared test for the difference in trends between males and females: $\chi^2=0.40$, $P=0.53$. | | | | |
| AMS score | | | | |
| 0 | 1 | | 1 | |
| 1 | 1.5 | (0.9-2.6) | 1.8 | (1.2-2.7)** |
| 2 | 4.9 | (2.3-10.4)*** | 2.5 | (1.3-4.5)** |
| 3 | 15.5 | (4.4-54.6)*** | 6.8 | (2.5-19.1)*** |
| 4&5 | 29.5 | (3.7-234.7)*** | ∞ | (2.1-∞)** |
| Chi-squared test for the difference in trends between males and females: $\chi^2_1=1.9$, $P=0.17$. | | | | |

*$P<0.05$. **$P<0.01$. ***$P<0.001$.

**Table VIII** Odds ratios and attributable proportions of melanoma in relation to phenotypical features

| | *Prevalence in cases (%)* | *Odds ratio (95% CI)* | *Attributable proportion (95% CI)* |
|---|---|---|---|
| One hundred or more naevi (relative to less than 100) | | | |
| Aged less than 40 | 28 | 4.4 (2.1-9.0) | 22% (8%-64%) |
| Aged more than 40 | 15 | 4.5 (2.1-9.4) | 12% (5%-32%) |
| Two or more AN (relative to less than 2) | | | |
| Aged less than 40 | 26 | 9.2 (3.6-22.4) | 23% (12%-43%) |
| Aged more than 40 | 14 | 10.8 (3.8-30.9) | 13% (4%-42%) |
| AMS score of ≥3 (relative to less than 3) | | | |
| Aged less than 40 | 21 | 16.1 (4.6-57.5) | 20% (5%-82%) |
| Aged more than 40 | 15 | 6.9 (2.9-16.6) | 13% (4%-36%) |
| Skin type I | | | |
| All ages | 12 | 3.2 (1.8-5.7) | 9% (4%-21%) |
| Skin type I or II and two or more atypical naevi | | | |
| All ages | 7 | 5.3 (2.8-20.5) | 6% (2%-19%) |

Augustsson et al. (1991) and Garbe et al. (1994) in Europe; Holly et al. (1987) in the USA and Nordlund et al. (1985) in Australia. In these studies, atypical or large numbers of common naevi were the strongest risk factor for melanoma with relative risks of the order of 10 for two or more atypical naevi. Whereas numbers of naevi are strongly associated with melanoma, less is known about the importance of the distribution of naevi in melanoma. Many studies have looked at sites such as the arms but there is little data on non-sun-exposed sites. Grob et al. (1990) reported that naevi on the buttocks were an important risk factor for melanoma cases with an odds ratio of 10.9 for five or more buttocks naevi. In one UK study, melanoma cases were more likely to have any naevi on the scalp than controls though their number (beyond one) were not counted (Swerdlow et al., 1986). Nordlund et al. (1985) found atypical naevi on non-sun-exposed sites such as the buttocks, the feet and the toe webs in melanoma cases in Australia, but the naevus counts at different body sites were not formally compared between cases and controls.

The presence of many atypical naevi was strongly associated with melanoma in the study reported here as were large number of common naevi. Naevus counts decreased with age in both cases and controls but older cases were more likely to have a large number of naevi than age-matched controls. The observation that the naevus count decreases with age is based on cross-sectional studies and it is not known to what extent this is owing to a tendency to larger numbers of naevi among more recent birth cohorts (Halpern et al., 1993). Atypical naevi were also found less commonly in the older study subjects. The proportion of melanoma attributable to the presence of two or more atypical naevi decreased with age; it accounted for a quarter of all melanomas below the age of 40 compared with 14% for the cases aged 40 years or above. A similar decrease in the aetiological fraction with age was seen for numbers of common naevi: 100 or more common naevi accounted for nearly twice as many melanomas in the cases aged below 40 years of age compared with cases older than 40. The differences in relative risk by age for naevus-related variables in our data may in part account for the differences in relative risks for these naevus variables between studies, which will have different weighting by age group in their all-age results.

It is not clear why atypical naevi and common naevi were more common in male than female cases and controls though melanoma is twice as common in females than males in the UK. The risk of melanoma associated with a large number of atypical naevi, however, was similar in each sex.

In this study, individuals with red hair or fair skin type were more likely to have atypical naevi. Weinstock et al. (1991) have reported that atypical naevi were more common in individuals with poor tanning ability. It is possible that atypical naevi are more easily expressed in individuals with fair skin because of their increased susceptibility to ultraviolet radiation. The presence of atypical naevi was associated with multiple melanoma primaries and this is consistent with observations in familial melanoma studies (Greene et al., 1985).

Naevi on unusual sites (dorsum of the feet, buttocks and anterior scalp) were risk factors for melanoma and remained significant after adjustment for atypical naevi. Iris naevi were associated with melanoma in this study. Rodriguez-Sains et al. (1986) reported that patients with AMS often had many iris naevi and suggested that their presence could be a marker of an expanded melanocytic system with numerous naevi found on the skin and in the eye. Albert et al. (1983) and Nordlund et al. (1985) had previously found that iris naevi were more common in cutaneous melanoma patients than in controls, although the numbers in the first study were small.

This study is the first to determine the risk of melanoma associated with the AMS phenotype in the general population rather than in the context of families at high risk. We used a scoring system for the AMS phenotype designed by ourselves, as there is no international agreement on the definition of this phenotype. This scoring system was originally designed for our family studies of melanoma (Newton et al., 1994). The AMS phenotype is thought to be a marker of a genetic susceptibility to melanoma, but sun exposure may influence the expression of this phenotype. There has been some controversy in the literature whether the AMS phenotype is a dichotomous or continuous phenotype (Traupe et al., 1989). The results of the present study favour a continuous phenotype, as the risk of melanoma increased with increasing AMS score. This would not favour the presence of a single autosomal dominant gene for the expression of this phenotype and it is more likely that several susceptibility genes are involved with the interaction of sun exposure or other environmental factors.

Thirty-two per cent and 16% of all melanomas in this study were attributable to an AMS score of 2 or 3 respectively and these aetiological fractions decreased with age. Among the five clinical features of the AMS phenotype scoring system, the presence of two or more atypical naevi yielded the highest relative risk but other clinical features of the phenotype were independently associated with an increased risk. None of these naevus characteristics were responsible for more than a quarter of all melanomas and it may be that for screening programmes in countries with a low incidence of melanoma, stronger predictors will be needed. However, by screening younger age groups, the AMS phenotype may have a role in the secondary prevention of melanoma.

### References

ALBERT DM, SEARL SS, FORGET B, LAVIN PT, KIRKWOOD J AND NORDLUND JJ. (1983). Uveal findings in patients with cutaneous melanoma. *Am. J. Ophthalmol.*, **95**, 474–479.

ALBERT LS, RHODES AR AND SOBER AJ. (1990). Dysplastic melanocytic nevi and cutaneous melanoma: markers of increased melanoma risk for affected persons and blood relatives. *J. Am. Acad. Dermatol.*, **22**, 69–75.

AUGUSTSSON A, STIERNER U, ROSDAHL I AND SUURKULA M. (1991). Common and dysplastic naevi as risk factors for cutaneous malignant melanoma in a Swedish population. *Acta Dermatol. Venereol. (Stockh)*, **71**, 518–524.

BLISS JM, FORD D, SWERDLOW AJ, ARMSTRONG BK, CRISTOFOLINI M, ELWOOD JM, GREEN A, HOLLY E, MACK T, MACKIE RM, OSTERLIND A, WALTER SD, PETO J AND EASTON DF. (1995). International Melanoma Analysis Group (IMAGE). Risk of cutaneous melanoma associated with pigmentation characteristics and freckling: systematic overview of ten case–control studies. *Int. J. Cancer*, **62**, 367–376.

COLEMAN MP, ESTEVE J, DAMIECKI P, ARSLAN A AND RENARD H. (1993). *Trends in Cancer Incidence and Mortality*. International Agency for Research on Cancer Scientific Publication 121. IARC: Lyon.

CLARK WH, REIMER RR, GREENE M, AINSWORTH AM AND MASTRANGELO MJ. (1978). Origin of familial malignant melanoma from heritable melanocytic lesions: the BK mole syndrome. *Arch. Dermatol.*, **114**, 732.

FITZPATRICK TB. (1988). The validity and practicality of sun reactive skin types 1 through 4. (Editorial). *Arch. Dermatol.*, **124**, 869–871.

GALLAGHER RP, ELWOOD JM AND HILL GB. (1986). Risk factors for cutaneous malignant melanoma in the Western Canada Melanoma Study. *Recent Results Cancer Res.*, **102**, 38–55.

GARBE C, BUTTNER P, WEISS J, SOYER HP, STOCKER U, KRUGER S, ROSER M, WECKBECKER J, PANIZZON R, BAHMER F, TILGEN W, GUGGENMOOS-HOLSMAN I AND ORFANOS CE. (1994). Risk factors for developing cutaneous melanoma and criteria for identifying persons at risk: mutlicenter case-control study of the Central Malignant Melanoma Registry of the German Dermatological Society. *J. Invest. Dermatol.*, **102**, 695-699.

GREENE MH, CLARK JR WH, TUCKER MA, KRAEMER KH, ELDER DE AND FRASER MC. (1995). High risk of malignant melanoma in melanoma-probe families with dysplastic naevi. *Ann. Int. Med.*, **102**, 458-465.

GREENLAND S. (1987). Variance estimators for attributable fraction estimates, consistent in both large strata and sparse data. *Stat. in Med.*, **6**, 701-708.

GROB JJ, GOUVERNET J, AYMAR D, MOSTAQUE A, ROMANO MH, COLLET AM, NOE MC, DISCONTANZO MP AND BONERANDI JJ. (1990). Count of benign melanocytic nevi as a major indicator of risk for nonfamilial nodular and superficial spreading melanoma. *Cancer*, **66**, 387-395.

HALPERN AC, DUPONT GUERRY, ELDER ED, WALLACE H, CLARK JR, SYNNESTVEDT M, NORMAN S AND AYERLE R. (1991). Dysplastic nevi as risk markers for sporadic non familial melanoma. A case-control study. *Arch. Dermatol.*, **127**, 995-999.

HALPERN AC, DUPONT-GUERRY IV, ELDER DE, TROCK B AND SYNNESTVEDT M. (1993). A cohort study of melanoma in patients with dysplastic nevi. *J. Invest. Dematol.*, **100**, 346S-349S.

HOLLY EA, KELLY JW, SHPALL SN AND CHIU SH. (1987). Number of melanocytic nevi as a major risk factor for malignant melanoma. *J. Am. Acad. Dermatol.*, **17**, 459-468.

INTERNATIONAL AGENCY FOR RESEARCH ON CANCER. (1992). *IARC Monographs on the Evaluation of Carcinogenesis to Humans. Vol. 55. Solar and Ultraviolet Radiation.* IARC: Lyon.

KOH HK, KLIGER BE AND LEW RA. (1990). Yearly review. Sunlight and cutaneous malignant melanoma: evidence for and against causation. *Photochem. Photobiol.*, **6**, 765-779.

NEWTON JA, BATAILLE V, GRIFFITHS K, SQUIRE JM, SASIENI P, CUZICK J, BISHOP DT AND SWERDLOW A. (1993). How common is the Atypical Mole Syndrome phenotype in apparently sporadic melanoma. *J. Am. Acad. Dermatol.*, **29**, 989-996.

NEWTON JA, BATAILLE V, PINNEY E AND BISHOP DT. (1994). Family studies in melanoma: identification of the Atypical Mole Syndrome (AMS) phenotype. *Mel. Res.*, **4**, 199-206.

NORDLUND JJ, KIRKWOOD J, FORGET BM, SCHEIBNER A, ALBERT DM, LERNER E AND MILTON GW. (1985). Demographic study of clinically atypical (dysplastic) nevi in patients with melanoma and comparison subjects. *Cancer Res.*, **45**, 1855-1861.

RODRIGUEZ SAINS RS. (1986). Ocular findings in patients with dysplastic nevus syndrome. *Ophthalmology*, **93**, 661-665.

SWERDLOW AJ, ENGLISH J, MACKIE RM, O'DOHERTY CJ, CLARK J AND HOLE DJ. (1986). Benign melanocytic naevi as risk factor for malignant melanoma. *Br. Med. J.*, **292**, 1555-1559.

TRAUPE H, MACHER E, HAMM H AND HAPPLE R. (1989). Mutation rate estimates are not compatible with autosomal dominant inheritance of the dysplastic nevus 'syndrome'. *Am. J. Med. Genet.*, **32**, 155-157.

TUCKER MA, GREEN MH, CLARK JR WH, KRAEMER KH AND FRASER MC. (1983). Dysplastic nevi in the scalp of prepubertal children from melanoma prone families. *J. Pediatr.*, **103**, 65-69.

WEINSTOCK MA, STRYKER WS, STAMPFER MJ, LEW RA, WILLETT WC AND SOBER AJ. (1991). Sunlight and dysplastic nevus risk. Results of a clinic-based control study. *Cancer*, **67**, 1701-1706.